# HiLDA: a statistical approach to investigate differences in mutational signatures

**DOI:** 10.7717/peerj.7557

**Published:** 2019-08-28

**Authors:** Zhi Yang, Priyatama Pandey, Darryl Shibata, David V. Conti, Paul Marjoram, Kimberly D. Siegmund

**Affiliations:** 1 Department of Preventive Medicine, Keck School of Medicine, University of Southern California, Los Angeles, CA, United States of America; 2 Department of Pathology, Keck School of Medicine, University of Southern California, Los Angeles, CA, United States of America

**Keywords:** Mutational signatures, Somatic mutation, Colorectal cancer, Latent dirichlet allocation, Deconvolution

## Abstract

We propose a hierarchical latent Dirichlet allocation model (HiLDA) for characterizing somatic mutation data in cancer. The method allows us to infer mutational patterns and their relative frequencies in a set of tumor mutational catalogs and to compare the estimated frequencies between tumor sets. We apply our method to two datasets, one containing somatic mutations in colon cancer by the time of occurrence, before or after tumor initiation, and the second containing somatic mutations in esophageal cancer by sex, age, smoking status, and tumor site. In colon cancer, the relative frequencies of mutational patterns were found significantly associated with the time of occurrence of mutations. In esophageal cancer, the relative frequencies were significantly associated with the tumor site. Our novel method provides higher statistical power for detecting differences in mutational signatures.

## Introduction

A variety of mutational processes occur over the lifetime of an individual, and thereby uniquely contribute to the catalog of somatic mutations observed in a tumor. Some processes leave a molecular signature: a specific base substitution occurring within a particular pattern of neighboring bases. A variety of methods exist to discover mutational signatures from the catalog of all somatic mutations in a set of tumors, estimating the latent mutational signatures as well as the latent *exposures* (i.e., fraction of mutations) each signature contributes to the total catalog. The first large study of mutational signatures in cancer identified variation in mutational signatures and mutational exposures across 21 different cancer types (Alexandrov et al., 2013b). To better understand the sources of variation in the mutational exposures across cancers, our interest is in statistical methods used to characterize these latent mutational exposures across different cancer subtypes. Moreover, by classifying mutations by their time of occurrence, before or after tumor initiation, we can investigate whether new mutational processes occur during tumor growth.

Previous studies interested in comparing mutational exposure estimates between different groups of tumor catalogs conducted a post hoc analysis (Cancer Genome Atlas Research Network, 2017; Chang et al., 2017; Hillman et al., 2017; Letouzé et al., 2017; Meier et al., 2018; Haradhvala et al., 2018; Qin et al., 2018; Olivier et al., 2019; Guo et al., 2018). The analysis proceeded in two stages. First, they performed one of the several different approaches for mathematically extracting the latent mutational signatures and their exposures from the mutational catalogs (see Baez-Ortega & Gori (2017) for a review of such methods). Later, they conducted an independent test of association between the point estimates of the mutational exposures and external covariates. Examples of covariates included cancer subtype, or patient history of alcohol or tobacco use. However, the variation of the exposure estimates is affected by two factors, the number of mutations in the tumor and the variation in exposure frequency in the patient population. The former, the number of mutations in the tumor, affects the accuracy of the exposure estimates. The application of the Wilcoxon rank-sum test on the exposure estimates does not take into consideration their accuracy, which can lead to loss of efficiency and test power. We address this by introducing a unified parametric model for testing variation of mutational exposures between groups of mutational catalogs, where the exposure frequencies are modeled using a Dirichlet distribution.

We propose a hierarchical latent Dirichlet allocation model (HiLDA) that adds an additional level to the latent Dirichlet allocation (LDA) model from Shiraishi et al. (2015). Shiraishi’s model, like the majority of deconvolution approaches, focuses on signatures for single-nucleotide substitutions, characterizing the mutation types by context, using local features in the genome such as the pattern of flanking bases and possibly the transcription strand. Other methods available to discover and characterize mutational signatures include the Wellcome Trust Sanger Institute (WTSI) Mutational Signature Framework, Emu, and signeR (Alexandrov et al., 2013b; Fischer et al., 2013; Rosales et al., 2016). Both WTSI Framework and signeR were developed based on the non-negative matrix factorization while Shiraishi’s model and Emu are probabilistic models using an expectation–maximization algorithm. However, all methods except for Shiraishi’s model describe three-base contexts in mutational signatures. For both model parsimony and interpretation, we choose to extend Shiraishi et al.’s LDA model. First, it requires fewer parameters than competing methods, giving it higher power to detect patterns five bases in length compared to other models that consider only three-base contexts (Shiraishi et al., 2015). For example, to capture the five-base context in a mutational signature, only requires 17 = (6 − 1) + 4 × (4 − 1) free parameters rather than 1,535 = 6 × 4^4^ − 1 when using the non-negative matrix factorization based method proposed by Alexandrov et al. (2013b). Second, signature visualization methods lead to easy interpretation; an example is the common C > T substitution at CpG sites instead of the more complicated NpCpG patterns that appear when using the trinucleotide context. Like the LDA model, HiLDA retains all the functionality for estimating both the latent signatures and the latent mutational exposure of each signature for each tumor catalog. Our newly-added hierarchical level allows HiLDA to simultaneously test whether those mean exposures differ between different groups of catalogs while accounting for the uncertainty in the exposure estimates. Additionally, we can now parse out differences in group means in the presence of differences in group variances, which is not tenable when using post hoc nonparametric location-scale tests.

In this paper, we use HiLDA to study mutational exposures in two cancer data sets, one colon and the second esophageal.

## Methods

### Hierarchical Bayesian Mixture Model

We introduce a hierarchical latent Dirichlet allocation model (HiLDA) using the following notation, also summarized in Table 1. Let *i* index the mutational catalog and *j* the mutation. The nucleotide substitutions are reduced to six possible types (C >A, C >T, C>G, T >A, T>C, T>G) to eliminate redundancy introduced by the complementary strands. Each observed mutation is characterized by a vector, ***X***_*i*,*j*_ describing the nucleotide substitution (e.g., C >T) and a set of genomic features in the neighborhood. Example features include the base(s) 3′ and 5′ of the nucleotide substitution (C, G, A, T), and the transcription strand (+, −). Each observed feature characteristic, *x*_*i*,*j*,*l*_ for mutation feature *l*, takes values in the set {1, 2, …, *M*_*l*_} (where *M*_*l*_ = 6 for the nucleotide substitution, or 4 for a flanking base, and 2 for the transcription strand).

**Table 1:** List of notation.

**Notation**	**Description**
*I*	Total number of mutational catalogs (indexed by *i*)
*J*_*i*_	Number of observed mutations in *ith* mutational catalog (indexed by *j*)
*L*	Number of features to include. Here, we use the nucleotide substitution, flanking bases and transcription strand (indexed by *l*)
***M***	Vector of the maximum numbers of possible values, (*M*_1_, …, *M*_*L*_), for each mutation feature, (indexed by *M*_*l*_), *M*_1_ = 6 for nucleotide substitution, *M*_2_ = 4 for flanking base, (A, C, G, T), *M*_*L*_ = 2 for transcription strand, (+, − )
*K*	Total number of mutational signatures (indexed by *k*)
***X***_*i*,*j*_	Observed mutation characteristic vector, (*x*_*i*,*j*,1_, …, *x*_*i*,*j*,*L*_), for the *jth* mutation from the *ith* mutational catalog (indexed by *x*_*i*,*j*,*l*_)
*z*_*i*,*j*_	Index of the latent assignment for ***X***_*i*,*j*_, *z*_*i*,*j*_ ∈ {1, …, *K*}
***q***_*i*,*k*_	Probability vector of signature *k* exposure in mutational catalog *i*, (*q*_*i*,1_, …, *q*_*i*,*K*_), with ∑_*k*_*q*_*i*,*k*_ = 1
***f***_*k*,*l*_	Probability vector of observing any of *M*_*l*_ elements for *lth* mutation feature, ***f***_*k*,*l*_ = (*f*_*k*,*l*,1_, …, *f*_*k*,*l*,*M*_*l*__) with ∑_*m*_*l*__*f*_*k*,*l*,*m*_*l*__ = 1
***F***_*k*_	A tuple of probability vectors with length *L*, (***f***_*k*,1_, …, ***f***_*k*,*L*_)
***g***	A vector indicating group membership of the samples. (*g*_*i*_ ∈ {1, 2} for each sample *i*)
*α*	A tuple of concentration parameters of a Dirichlet distribution with length *K*, (*α*_1_, …, *α*_*K*_), where the dispersion *ϕ* = ∑_*k*_*α*_*k*_
*μ*	A tuple of expected values of ***q*** of a Dirichlet distribution with length *K*, (*μ*_1_, …, *μ*_*K*_), where ∑_*k*_*μ*_*k*_ = 1.

We assume each mutation belongs to one of *K* distinct signatures. A specific mutational signature *k* is defined by an *l*-tuple of probability vectors, ***F***_*k*_, denoting the relative frequencies of the *M*_*l*_ discrete values for the *l* features, i.e., a vector ***f***_*k*,*l*_ for the *M*_*l*_ values corresponding to feature *l*. We let *z*_*i*,*j*_ denote the unique latent assignment of mutation ***X***_*i*,*j*_ to a particular signature. Then, given the signature to which a mutation belongs, the probability of observing a mutational pattern is calculated as the product of the mutation feature probabilities for that signature. Thus, for signature *k* we write *Pr*(***X***_*i*,*j*_|*z*_*i*,*j*_) = ∏_*l*_*f*_*k*,*l*_(*x*_*i*,*j*,*l*_|*z*_*i*,*j*_). This assumes independent contributions of each feature to the signature. To model each multinomial distribution of ***f***_*k*,*l*_, we use a non-informative Dirichlet prior distribution with all concentration parameters equal to one.

The unique personal exposure history of each individual leads to them having a particular (latent) vector, ***q***_*i*_, indicating the resulting contribution of each of the *K* signatures to that individual’s mutational catalog. These ***q***s are modeled using a Dirichlet distribution with concentration parameters *α*, i.e., ***q***_*i*_ ∼ *Dir*(*α*). Extending this model to the two-group setting, we allow the Dirichlet parameters to depend on group, *Dir*(*α*^(*g*_*i*_)^), with *g*_*i*_ indexing the group corresponding to the *i*th catalog (*g*_*i*_ = 1 or 2). The mean mutational exposures, *E*(***q***_*i*_), denoted by *μ*^(*g*_*i*_)^, are represented by using the concentration parameters, i.e., *μ*^(*g*_*i*_)^ = *α*^(*g*_*i*_)^∕∑*α*^(*g*_*i*_)^.

With this extension, we can infer differences in mutational processes between groups of catalogs by testing whether the mean mutational exposures differ between the two sets, i.e., at least one *μ*_*k*_^(1)^ ≠ *μ*_*k*_^(2)^. The likelihood and prior of the multi-level model is specified as follows,

\begin{eqnarray*}{x}_{i,j,l}{|}{z}_{i,j}& \sim Multinomial({\mathbi{f}}_{{z}_{i,j},l}) \end{eqnarray*}

\begin{eqnarray*}{z}_{i,j}& \sim Multinomial({\mathbi{q}}_{i}{|}g) \end{eqnarray*}

\begin{eqnarray*}{\mathbi{q}}_{\mathbi{i}}{|}{g}_{i}& \sim Dir({\alpha }^{({g}_{i})}) \end{eqnarray*}

For full details see See Text S1 and Fig. S1.

### Testing for differences in signature exposures

To characterize the signature contributions for different sets of tumor catalogs, we wish to conduct a hypothesis test that there is no difference in mean exposures versus the alternative that the mean exposure of at least one signature differs between the two groups, i.e., *H*_0_:*μ*^(1)^ = *μ*^(2)^ vs. *H*_1_: at least one *μ*_*k*_^(1)^ ≠ *μ*_*k*_^(2)^. We propose both local and global tests, implemented in a Bayesian framework. The former provides signature-level evaluations to determine where the differences in mean mutational exposures occur, while the latter provides an overall conclusion about any difference in mean mutational exposures. The details of our implementation are given in our Just Another Gibbs Sampler (JAGS) scripts and Source code is freely available in GitHub at https://github.com/USCbiostats/HiLDA (Plummer, 2003).

#### A local test to identify signatures with different exposures

We propose a signature-level (local) hypothesis test to allow us to infer which signature(s) contribute a different mean exposure to the mutational catalogs across tumor sets, i.e., *μ*_*k*_^(1)^ ≠ *μ*_*k*_^(2)^. To measure the difference between mean signature exposure vectors, we implement HiLDA by specifying two Dirichlet distributions, *Dir*(*α*^(1)^) and *Dir*(*α*^(2)^), as priors for the distribution of mutational exposures ***q***_*i*_ of each group (Spiegelhalter et al., 2003). Using this formulation, the difference between the two groups of the mean exposure of signature *k* is calculated as,

(1) \begin{eqnarray*}{\Delta }_{k}={\mu }_{k}^{(2)}-{\mu }_{k}^{(1)}= \frac{{\alpha }_{k}^{(2)}}{\sum _{k}{\alpha }_{k}^{(2)}} - \frac{{\alpha }_{k}^{(1)}}{\sum _{k}{\alpha }_{k}^{(1)}} .\end{eqnarray*}

For all parameters, ${\alpha }_{k}^{(1)}$’s and ${\alpha }_{k}^{(2)}$’s, we use independent, non-informative gamma distribution priors with a rate of 0.001 and shape of 0.001. Since JAGS suffers from convergence issue when estimating parameters very close to zeros, we truncate that distribution to be ≥0.05. This results in an approximate mean of 107.7 and an approximate variance of 9.62 × 10^4^.

We estimate parameters via Markov chain Monte Carlo (MCMC) using two chains (Carlin & Chib, 1995). We assess convergence of the two MCMC chains using the potential scale reduction factor (Rhat) in Gelman & Rubin (1992), which is required to be less than or equal to 1.05 for all parameters in order to conclude that the MCMC run has converged. After obtaining the posterior distribution of the differences (i.e., of Δ_*k*_), there are two possible approaches to performing inference. We can: (1) use the Wald test to compute the *P*-value using the means and standard errors of the posterior distribution for Δ_*k*_; (2) determine whether the 95% credible interval of the posterior distribution for Δ_*k*_ contains zero.

#### A global test using the Bayes factor

We also propose a global test to provide an overall conclusion on whether the mean exposures differ between groups of catalogs. It uses the Bayes factor, the ratio of posterior to prior odds in favor of model *H*_1_ (*H*_1_: at least one ${\mu }_{k}^{(1)}\not = {\mu }_{k}^{(2)}$, *k* = 1, …, *K*) compared to model *H*_0_ (*H*_0_: *μ*^(1)^ = *μ*^(2)^), to indicate the strength of evidence that they do differ, without explicit details on how they differ. Thus, we can calculate the Bayes factor as:

(2) \begin{eqnarray*}Bayes~Factor= \frac{Pr({H}_{1}{|}Data)}{Pr({H}_{0}{|}Data)} \left/ \frac{Pr({H}_{1})}{Pr({H}_{0})} . \right. \end{eqnarray*}

Since the likelihood is analytically intractable, the Bayes factor is calculated via MCMC (Carlin & Chib, 1995). In order to estimate the Bayes factor, during the MCMC analysis, a single binary hypothesis index variable is used to indicate which hypothesis explains the observed data (Lodewyckx et al., 2011). The parameters of two Dirichlet distributions, *Dir*(*α*^(1)^) and *Dir*(*α*^(2)^), are drawn from the same prior if the index takes the value 1, whereas they are drawn from different priors if it takes the value 2. Initially, the prior hypothesis odds is set to be 0.5/0.5 = 1, which means that both hypotheses are assumed equally likely under the prior. In order to improve computational efficiency in extreme situations in which one hypothesis dominates the other, we can use a different prior odds value (Carlin & Chib, 1995). A Bayes Factor (BF) between 3–10 indicates substantial support for the model with different mean exposures in the two groups (*H*_1_) (Jeffreys, 1998). A BF > 10 indicates strong support.

### Two-stage inference methods using the point estimates of mutational exposures

An alternative approach is to perform hypothesis testing using point estimates of the mutational exposures, ${\hat {\mathbi{q}}}_{i}$, in a two-stage analysis, which we refer to as the “two-stage” method (TS). We used the R package **pmsignature** to estimate $\hat {\mathbi{q}}$ (Shiraishi et al., 2015). Other methods are also available, but we selected **pmsignature** for the purpose of comparisons to the results from HiLDA since it assumes the same model for estimating signatures under independence of features. We summarize the steps of the TS method as follows:

1. Jointly estimate the vectors of mutational signature exposures, ***q***_*i*_, for each mutational catalog.
2. Test for differential mutational exposures for signature *k* by performing the Wilcoxon rank-sum test on the $\hat {{\mathbi{q}}_{k}}$.

However, we note that the Wilcoxon rank-sum test in stage 2 is also sensitive to changes in variance across the two groups, which might lead to significant results even when there has been no change in mean exposures (Kasuya, 2001; Ruxton, 2006). We implemented the two-stage method using R version 3.5.0 (R Core Team, 2017). A two-sided *P* value of less than 0.05 was considered statistically significant.

### Choosing the number of signatures

The number of signatures, *K*, needs to be determined prior to any of the above analyses. We adopted the method of Shiraishi et al. (2015) to determine *K*. Their method is based on the following criteria:

1. The optimal value of *K* is selected over a range of *K* values such that the likelihood remains relatively high while simultaneously having relatively low standard errors for the parameters.
2. Pairwise correlations between any two signatures (the *k*th signature and the *k*′th signature, say) are measured by calculating the Pearson correlation between their estimated mutational exposures across all samples, (i.e., the correlation between $({\hat {q}}_{1,k},\ldots ,{\hat {q}}_{I,k})$ and $({\hat {q}}_{1,{k}^{{^{\prime}}}},\ldots ,{\hat {q}}_{I,{k}^{{^{\prime}}}})$). *K* is chosen such that no strong correlation (i.e., >0.6) exists between any pair.

For full details see Shiraishi et al. (2015).

### Application

#### USC colon cancer data

Our goal is to identify whether any new mutational signatures occur during colon cancer growth that distinguish cancer evolution from normal tissue evolution. To achieve this, we classify somatic mutations into two catalogs according to time of occurrence: those that accumulated between the time of the zygote and the first tumor cell, which we call trunk mutations, and those that occur *de novo* during tumor growth, which we refer to as branch mutations. We then estimate mutational signatures in the two sets of catalogs and test whether the mean mutational exposures differ between them.

We analyzed a total of 16 colon tumors. Tumor and adjacent normal tissue were subject to whole exome sequencing, and somatic mutations called using the GATK pipeline and MuTect (details below). Somatic mutations in the tumors were defined as nucleotide variants that were detected in tumor tissue but did not also appear in the patient-matched normal tissue. We used multi-region tumor sampling to allow us to distinguish between trunk from branch mutations (Siegmund & Shibata, 2016). Each tumor was sampled twice, with bulk tissue samples taken from opposite tumor halves. We classified somatic mutations appearing in both tumor halves as trunk, because only trunk mutations are likely to appear in both tumor halves, while mutations found on only one side of a tumor were labeled as branch. This approach has previously been shown to be 99% sensitive for calling trunk mutations and 85% sensitive for calling branch mutations (Siegmund & Shibata, 2016). Fifteen of the 16 tumors were previously analyzed in a study of cell motility (Ryser et al., 2018).

The sequence data were processed using the GATK pipeline version 3.7 (DePristo et al., 2011) and somatic mutations called with MuTect version 1.1.7 (Cibulskis et al., 2013), applying the quality filters KEEP (default parameters) and COVERED (read depth of 14 in tumor and 10 in matched normal - use of a lower coverage threshold in normal tissue is as recommended in Cibulskis et al. (2013)). We excluded any mutations that either had an allele frequency less than 0.10, because sequencing errors are more common among low-frequency mutations (Cibulskis et al., 2013), or that were not also found by Strelka (Saunders et al., 2012), which we used as a confirmatory control. Somatic mutations on chromosomes 1 to 22 were used for mutational signature analysis. Our final data set is available for download from https://osf.io/a8dzx/.

#### Esophageal Adenocarcinoma (EAC) data

Here, we test for possible group differences in esophageal adenocarcinoma mutational exposures by four clinically important covariates. In papers by Alexandrov et al. (2013a) and Shiraishi et al. (2015), 146 tumor samples of esophageal cancer patients from Dulak et al. (2013) were analyzed to extract mutational signatures. We downloaded the somatic mutations for this analysis from (ftp://ftp.sanger.ac.uk/pub/cancer/AlexandrovEtAl/somatic_mutation_data/Esophageal/). Information for the four clinical variables were retrieved from cBioPortal (https://www.cbioportal.org/study/summary?id=esca_broad) (Cerami et al., 2012; Gao et al., 2013; Dulak et al., 2013). We extended the analysis of Shiraishi et al. (2015), applying HiLDA to test whether the mutational exposures of the four signatures they found differ by sex (120 male vs. 25 female), age group (120 ≥ 60 years vs. 25 < 60 years), smoking status (47 smokers vs. 19 non-smokers), or tumor site (41 esophagus vs. 52 cardia/gastric-esophageal junction(GEJ)).

## Results

### Tumor evolution in USC colon cancer data

A total of 12,554 somatic single-nucleotide substitutions were identified, with a median of 277 per sample (range: 82–1,762) (see Table S1). One tumor with microsatelite instability has more than double the number of somatic mutations (1,751 side A, 1,762 side B) than any of the remaining 30 catalogs (all <750 mutations). In our first analysis, we compared the mutational exposures in side A to those in side B. If the tumors represent a single clonal expansion, we would expect similar mutational exposure frequencies in the two catalogs from the same tumor. Indeed, this is what we found (Table 2).

**Table 2:** Comparing mutational exposures from two sets of mutational catalogs, Side A and Side B, in the USC data.

	Side A–Side B	HiLDA-CI	HiLDA-Wald	TS-Wilcoxon
Tests^a^	**Coef**.	[95% C.I.]^b^	*p* value	*p* value
Δ_1_	0.002	[−0.079, 0.083]	0.986	0.780
Δ_2_	0.000	[−0.029, 0.029]	0.988	0.897
Δ_3_	−0.002	[−0.083, 0.086]	0.961	0.985
*H*_0_:Δ_1_ = Δ_2_ = Δ_3_ = 0		Bayes Factor_*M*_2_∕*M*_1__ = 0.021		

**Notes.**

a ${\Delta }_{k}= \frac{{\alpha }_{k}^{(2)}}{{\sum }_{k}{\alpha }_{k}^{(2)}} - \frac{{\alpha }_{k}^{(1)}}{{\sum }_{k}{\alpha }_{k}^{(1)}} $, the difference in the mean exposure of signature *k* in group 1 and 2.

b 95% credible interval from the posterior distribution.

**Figure 1:**
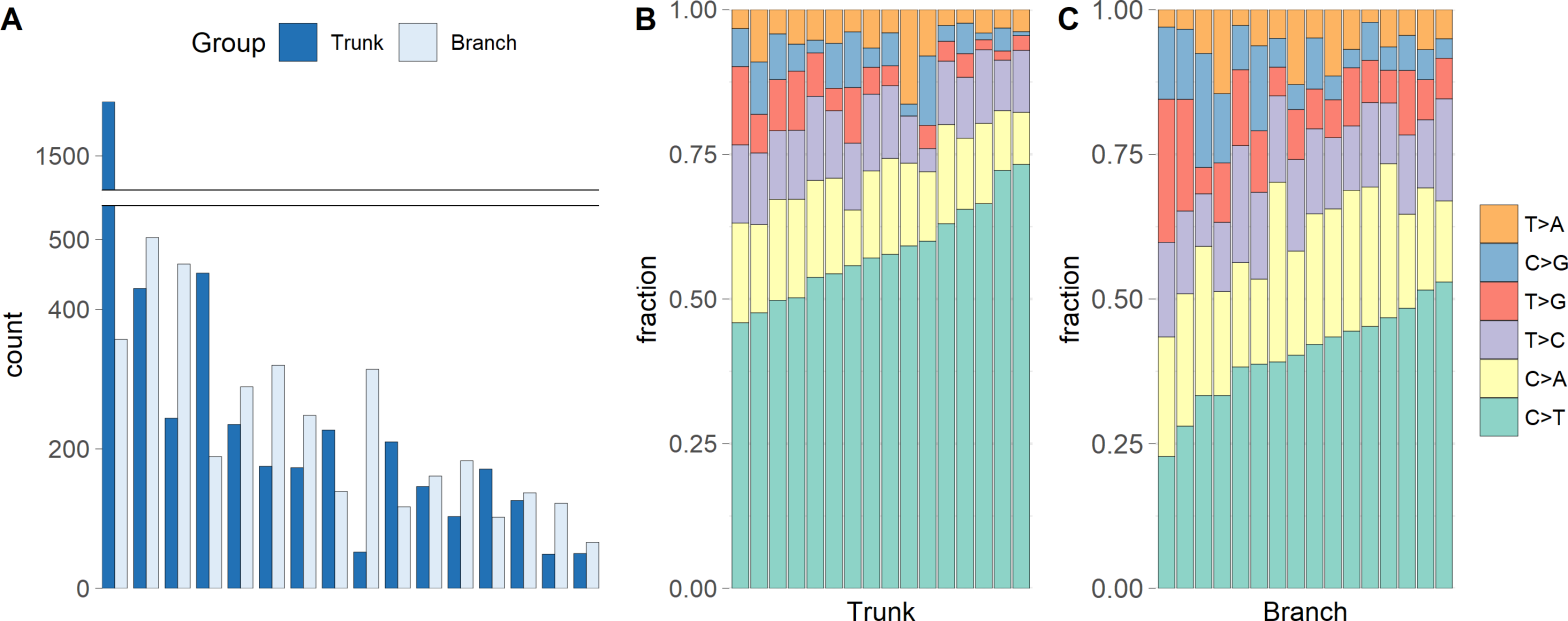
The numbers of somatic mutations in 32 mutational catalogs obtained from 16 colon cancer patients in the USC data and their mutation spectra. (A) The number of somatic mutations in 16 tumors, each of which contributes two mutational catalogs denoted as trunk (dark blue) and branch (light blue). (B) The percentage bar plot of relative frequencies for six substitution types in 16 trunk mutational catalogs. (C) The percentage bar plot of relative frequencies for six substitution types in 16 branch mutational catalogs.

We identified a median of 174 trunk and 186 branch mutations per tumor. The numbers ranged from 49 to 1,578 trunk mutations and from 66 to 503 branch mutations (Fig. 1A). Interestingly, the microsatellite instable tumor had the most trunk mutations, but not the most branch mutations, suggesting that during tumor growth the mutation frequency is similar in microsatellite stable and instable tumors. Figs. 1B and 1C show that the C >T substitution is most common in all trunk catalogs, and most branch catalogs. The spontaneous deamination of methylated Cs in CpGs is known to contribute to hotspots of C >T mutation in the genome.

**Figure 2:**
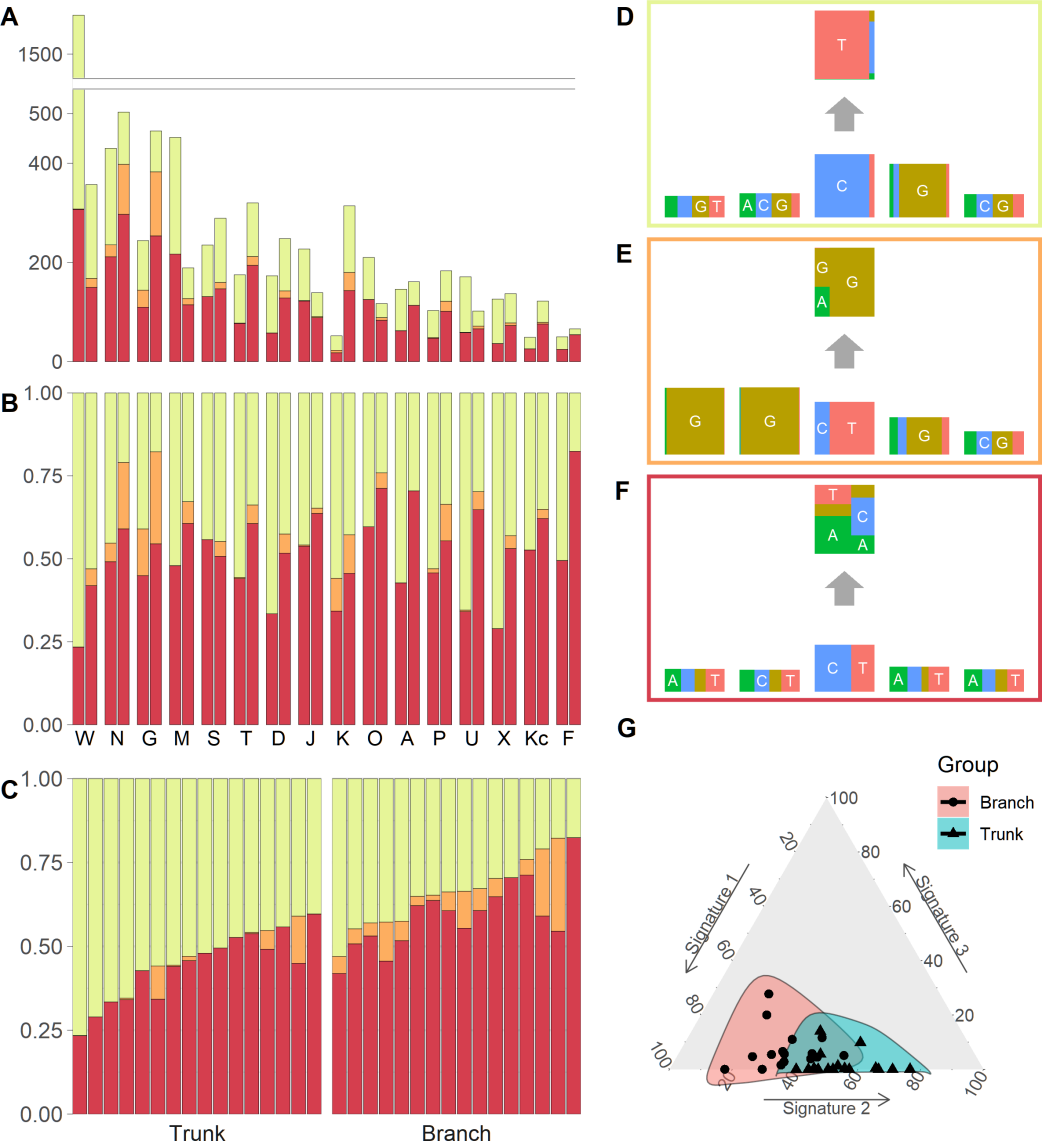
Mutational exposures and three mutational signatures from the analysis of 16 trunk mutational catalogs and 16 branch mutational catalogs in the USC data (16 colon cancer patients). (A) Barplot of the somatic mutation counts, by signature type, sorted in a descending order of the total number of mutations. Each grouped pair contain the trunk mutations and the branch mutations. *y*-axis shows total number of mutations. (B) Barplot of the somatic mutation counts, again by signature type and sorted in a descending order of the total number of mutations. Again, each grouped pair contains the trunk mutations and the branch mutations, but now the *y*-axis is rescaled to show proportions rather than total mutation count. (C) The same data as in Fig. 2B, but now separate into trunk and branch mutations. Within each group the plots are sorted by the exposure frequency of the first signature (yellow). (D) The yellow mutational signature with four flanking bases. (E) The orange mutational signature with four flanking bases. (F) The red mutational signature with four flanking bases. (G) The distributions of mutational exposures of the three mutational signatures highlighted by group, where the branch mutational catalogs are highlighted as pink and the trunk ones are highlighted as blue.

We identified three mutational signatures in our data (see Fig. S2). Those three signatures, and their corresponding exposures, are depicted in Figs. 2A, 2B and 2C. For each mutational signature, we compute the probabilities for the 1536 possible five-base signature patterns by taking the product of the feature component probabilities. We use these multinomial vectors to calculate the cosine similarity between pairs of signatures (Yang et al., 2019). The signature shown in the yellow box in Fig. 2D, involving C >T mutations at CpG sites, resembles signature 7 in Shiraishi et al. (2015) (cosine similarity 0.95), where it was identified in 25 out of 30 cancer types and likely relates to the deamination of 5-methylcytosine (‘aging’); the signature in the orange box in Fig. 2E, involving T>G mutations at GpGpTpG sites, is novel; the third signature, in the red box in Fig. 2F, is most similar to signature 23 in Shiraishi et al. (2015) (cosine similarity 0.85), where it was identified in four other cancer types. The pairwise cosine similarities between pairs of our yellow, orange and red signatures are 0.12, 0.01, and 0.02 which are rather dissimilar from each other given the [0, 1] range for cosine similarity. Using HiLDA, we test whether the three signatures differ in mean exposure between trunk and branch mutations.

Our global test strongly suggests that, in our data, the signature exposures differ between trunk and branch catalogs (Bayes Factor = 1265.0). A Bayes Factor greater than 10 is considered strong evidence for model *H*_1_ (Jeffreys, 1998). Each of the individual signatures (depicted in Figs. 2D, 2E and 2F) is found to differ in exposure between the two sample groups, a conclusion supported by both HiLDA and the two-stage method (Table 3). From Fig. 2C, it is evident that the exposures of the first (‘aging’) signature in trunk mutations is almost always greater than that for the matching catalog of branch mutations, which is intuitively consistent with the fact that trunk mutations may well reflect an accumulation of mutations over the life of the subject, whereas branch mutations are accumulated only after tumor initiation. For the previously unseen signature, the higher exposures in branch catalogs might suggest that this signature’s underlying mechanism for generating mutations might be associated with the processes occurring during tumor evolution as opposed to normal development. From Fig. 2G, we observed that the distributional ranges of the two groups of mutational exposures have some overlaps, but that the centers of each group, i.e., the means of mutational exposures, are clearly deviated from each other. However, the distributional radii, indicating the variances of mutational exposures, do not substantially differ between the groups.

**Table 3:** Comparing mutational exposures in colorectal cancer from two sets of mutational catalogs, trunk and branch, in the USC data.

	Branch-Trunk	HiLDA-CI	HiLDA-Wald	**TS-Wilcoxon**
**Tests**^a^	**Coef**.	[95% C.I.]	*p* value	*p* value
Δ_1_	−0.210	[−0.295, −0.127]	<0.0001	0.0002
Δ_2_	0.064	[0.035, 0.099]	0.0001	0.0075
Δ_3_	0.146	[0.056, 0.231]	0.0011	<0.0001
*H*_0_:Δ_1_ = Δ_2_ = Δ_3_ = 0		Bayes Factor_*M*_2_∕*M*_1__ = 1265.0		

**Notes.**

a ${\Delta }_{k}= \frac{{\alpha }_{k}^{(2)}}{{\sum }_{k}{\alpha }_{k}^{(2)}} - \frac{{\alpha }_{k}^{(1)}}{{\sum }_{k}{\alpha }_{k}^{(1)}} $, the difference in the mean exposure of signature *k* in group 1 and 2.

b 95% credible interval from the posterior distribution.

We sought to validate the discovery of the previously unseen signature by repurposing targeted sequencing data from the same tumor set (Siegmund & Shibata, 2016) and using publicly available data from the Cancer Genome Atlas. Four T>G substitutions that we assigned to the previously unseen signature were part of a much larger independent validation set of mutations subjected to targeted, high-coverage Ampliseq technology (Siegmund & Shibata, 2016); all four of these T>G substitutions failed to validate. Further, a systematic analysis of data from the Cancer Genome Atlas also did not find evidence for this signature (Williams et al., 2016). Therefore, we cannot rule out that the signature is the result of sequencing error.

### Esophageal adenocarcinoma

**Figure 3:**
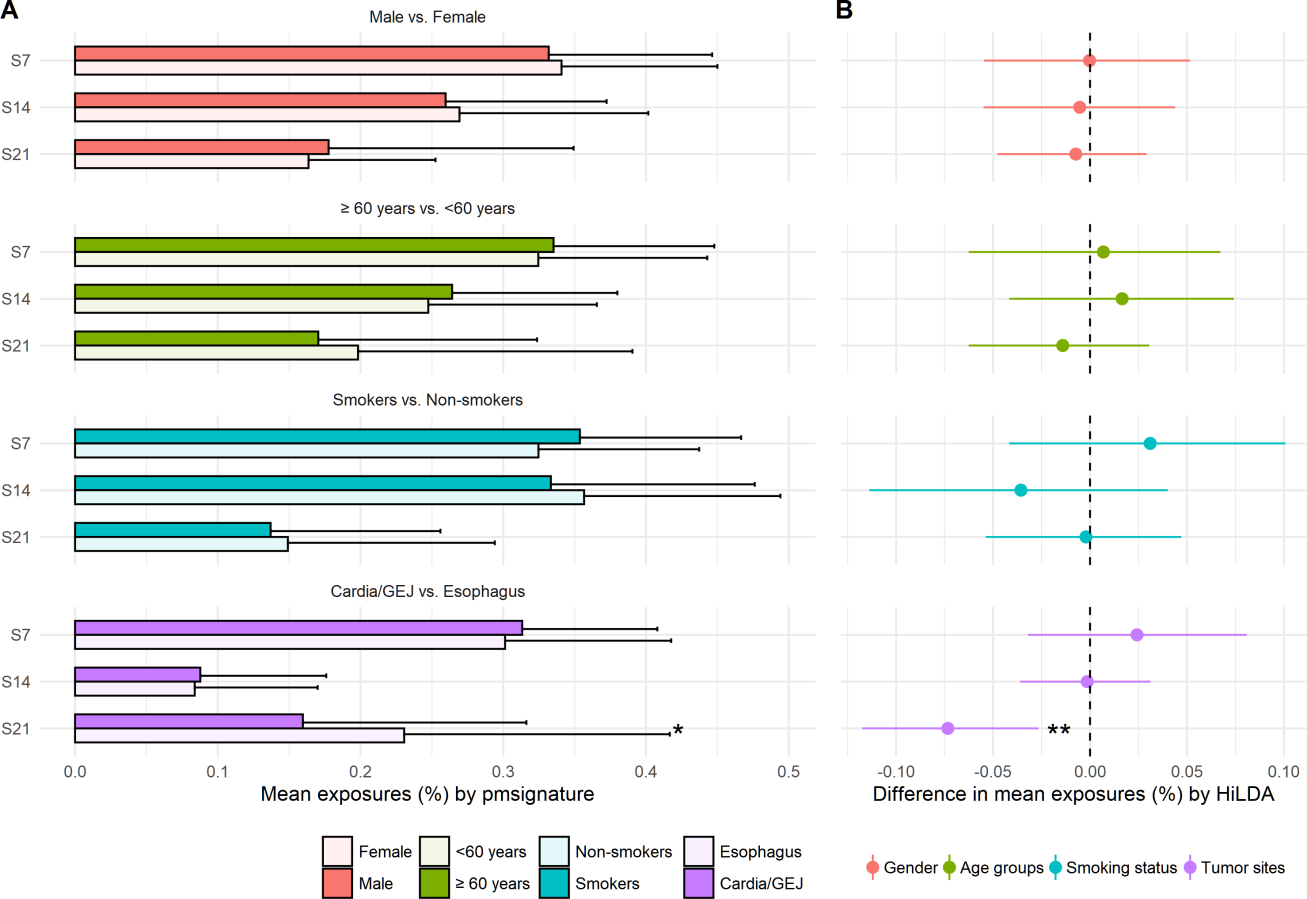
Estimated mutational exposures and posterior distributions of mean differences in mutational exposures from the analysis of the EAC data (146 esophageal adenocarcinoma patients). (A) Barplot of mean mutational exposures of three signatures by sex, age groups, smoking status, and tumor sites derived from pmsignature. The significance level of TS approach is denoted by asterisks (**, <0.005; *, <0.05). The mutational exposures do not sum to one since the frequency of remaining mutations (those not assigned to these three signatures) is not displayed. (B) 95% credible interval of mean differences in mutational exposures of four signatures derived from HiLDA-CI with the significance level of HiLDA-Wald test. (**, <0.005; *, <0.05). The difference in mean exposures from HiLDA can differ from those estimated by pmsignature due to the covariate distribution in the hierarchical model.

We reanalyzed the 146 EAC previously studied by Shiraishi et al. (2015) and recovered the same four mutational signatures, C >T at CpG (S7), C >T or A at TpC (S14), T >G or C at Cp(T >G/C)pT (S21), and a signature capturing the remaining mutations, i.e., those that do not fall into the previous three signatures. We tested for differences in mutational exposures by sex, age, smoking status, and tumor site. Only tumor site showed some evidence of differences in mutational exposure by patient subgroup (Fig. 3). The TS approach showed the mutational exposure for signature S21 was lower in the cardia/GEJ compared to the esophagus (*p* = 0.019) (Fig. 3A). HiLDA only identified a significant deficit in the mutational exposure for S21 in the cardia/GEJ location (−7.3% with 95% credible interval: [−11.8%, −2.7%]; HiLDA-Wald *p* = 0.002) (Fig. 3B). However, the HiLDA global test showed no strong evidence for associations between mutational exposures and any of the four clinical variables age, sex, smoking status or tumor site (all Bayes Factors < 1), suggesting the differences with tumor site may not be real. Still, both HiLDA-CI and HiLDA-Wald tests return significant results even when using the Bonferroni method to adjust for multiple comparisons (−7.3% with 98.75% credible interval: [−12.9%, −1.5%]; adjusted *p* = 0.019). See Fig. S3 for more details. We now go on to assess the reliability of results using a simulation study.

### Simulation study

We conducted a simulation study to assess the performance of both HiLDA and the two-stage approach in terms of the false-positive rate (FPR) and true-positive rate (TPR), in local, univariate tests of the difference in mean exposure between two groups of mutational catalogs. We assess the functionality of the methods in a setting similar to that of the USC data, simulating somatic mutations directly using the estimated signatures (***f***_*k*_) from Figs. 2D, 2E and 2F for the same number of mutational catalogs (two groups of 16 catalogs each) and somatic mutations per catalog (*J*_*i*_ in Table S1). The mutational exposures (***q***_***i***_) were indirectly used to derive the concentration parameters of the Dirichlet distributions. The scenarios are as follows:

1. The two groups of mutational catalogs are from separate Dirichlet distributions with parameters *α*^(1)^ = (9.2, 0.2, 7.5) and *α*^(2)^ = (4.2, 0.6, 7.3). Here, the *αs* corresponds to the maximum-likelihood estimated parameters from the three exposure distributions in the trunk and branch mutational catalogs. This gives mean exposures of *μ*^(1)^ = (0.54, 0.01, 0.44) and *μ*^(2)^ = (0.35, 0.05, 0.60) in trunk and branch catalogs, respectively, for the aging signature, new signature, and random signature.
2. The two groups of mutational catalogs are from the same Dirichlet distribution, *Dir*(4.2, 0.6, 7.3), (so here we use the concentration parameters estimated from the branch mutational catalogs).

For each tumor, mutational exposures ***q***_***i***_, are drawn from the Dirichlet distribution. Each set of probabilities parameterize a multinomial distribution later used to probabilistically choose the underlying mutational signature for a mutation (See Fig. S4). Then, every mutation feature in the mutational pattern of the mutation is simulated independently from a corresponding multinomial distribution of the chosen signature. To estimate the FPRs, 1,000 sets of data were simulated for scenario 2, when there is no difference in the exposure distribution between two groups of mutational catalogs. The two-stage method is slightly conservative for 1st and 3rd signatures (resulting FPRs of 4.3%, 5.2%, and 4.3%) when testing at the 5% significant level (Table 4). In comparison, HiLDA showed better control of the FPR by using the 95% credible interval of the posterior distributions (4.8%, 5.0%, and 5.1%). The Wald test also showed control of the FPR, except in the case of the rare signature when it was noticeably lower (3.7%), presumably due to the asymmetric posterior distribution.

**Table 4:** The false positive rates (FPR), true positive rates (TPR), and updated true positive rates of both the two-stage method and HiLDA. The false positive rates (*n* = 1,000) and true positive rates (*n* = 200) of both the two-stage method and HiLDA when applied to the simulated data.

	**Methods**	Δ_1_	Δ_2_	Δ_3_
**FPRs**	HILDA-CI^a^	4.8%	5.0%	5.1%
	HILDA-Wald^b^	5.1%	3.7%	5.4%
	TS-Wilcoxon	4.3%	5.2%	4.3%
**TPRs**	HILDA-CI	99.5%	85.5%	91.5%
	HILDA-Wald	99.5%	80.5%	92.5%
	TS-Wilcoxon	99.0%	77.5%	88.0%

**Notes.**

a Percentage of 95% credible intervals that exclude zero.

b Percentage of *P*-values <0.05 after applying the Wald test to the posterior distribution.

We then moved to scenario 1, where we simulated 200 data sets with a difference in mean exposures between the two groups of catalogs. Here, the statistical powers of both HiLDA and the two-stage method are high when detecting the difference in exposures for the 1st and 3rd signatures (Table 4). In contrast, for the 2nd signature, which has the lowest mean mutational exposure, the TPRs of all methods are lower (77.5%–85.5%). By using the 95% credible interval of posterior distributions, HiLDA is able to distinguish a difference more often than the two-stage method (99.5% vs. 99.0%, 85.5% vs. 77.5%, and 91.5% vs. 88.0%). At the same time, using the credible interval resulted in higher TPRs compared to performing a Wald test (85.5% vs. 80.5% for the 2nd signature). In summary, across tests involving these three mutational signatures, HiLDA provides higher statistical power to the TS method with a tendency of better improvement for signatures with lower mutational exposures, i.e., the power difference between HiLDA and the TS method is the highest (8%) for signature 2 with the lowest mean mutational exposures. The improvements in the power to detect the mean exposure difference is presumably due to the fact that HiLDA accounts for the uncertainty in the estimated mutational exposures and provides better model fit of the posterior distributions. All data were simulated in R 3.5.0 using the hierarchical Bayesian mixture model described in the methods section. All replicates reached convergence with an Rhat value less than 1.05 for each of the scenarios shown in Tables 2–4.

## Discussion

In this paper, we present a new hierarchical method, HiLDA, that allows the user to simultaneously extract mutational signatures and infer mutational exposures between two different groups of mutational catalogs, e.g., trunk and branch mutations in our colon cancer application. Our method is built on the approach of Shiraishi et al. (2015), in which mutational signatures are characterized under the assumption of independence, and it is the first to provide a unified way of testing whether mutational processes differ between groups (here, between early and late stages of tumor growth). As a result, our method allows us to appropriately control the false positive rates while providing higher power by accounting for the accuracy in the estimated mutational exposures.

In our analysis of the USC data, which consist of 32 mutational catalogs extracted from tumors from 16 CRC patients, our method detected three signatures and indicated a statistically significant difference in mean exposures between groups. Two of the three signatures resemble signatures S7 and S23 found by Shiraishi et al. (2015). But, in addition, we found a novel signature. Signature 7 appears significantly more often in trunk mutations, which is consistent with the fact that it has previously been related to aging and trunk mutations have a longer time over which to occur (conceivably over the lifetime of the patient) than do branch mutations (which occur only during tumor growth). The new signature, which occurred more often in low frequency branch mutations, is very similar to a sequencing artifact described by Alexandrov et al. (2018) (cosine similarity = 0.93). We note that, for the USC data, the conclusions obtained from HiLDA were qualitatively the same as those obtained from the TS method. This is likely due to the relatively large effect size here (i.e., the difference of mean exposures between the two groups, divided by the standard errors of same, also known as the signal-to-noise ratio).

In the analysis of the EAC data using HiLDA, we detected a statistically significant increase in the mutational exposure of S21, which is consistent with the findings of excessive fraction of A(A >C) mutations in esophagus compared to cardia/GEJ found in Dulak et al. (2013). To explain, since mutational signatures features are defined in terms of substitutions by the pyrimidine (T and C), an A(A >C) transversion is equivalent to a (T >G)T transversion associated with S21. Also, we found that S21 greatly resembles Signature 17 published in Alexandrov et al. (2013b) (cosine similarity = 0.96), the hallmark signature of EAC that has been proposed to arise from oxidative damage due to gastrointestinal reflux (Nones et al., 2014). Alexandrov et al.’s Signature 17 has been shown to have a higher number of mutations in EAC compared to stomach cancers, which reinforces our results showing higher mutational exposures for S21 in tumors occurring in the tubular esophagus compared to those in the GEJ (Alexandrov et al., 2018). By comparing different testing results, it seems that both HiLDA-CI and HiLDA-Wald tests are more sensitive compared to the TS approach in detecting the difference. However, the global test, based on the Bayes factor, disagrees with the local test in the EAC data which might suggest that more samples are needed for the global test to sufficiently support model *H*_1_.

In the simulation study, both HiLDA and the TS approach were applied to datasets consisting of 16 tumors simulated under two scenarios to test for between group differences in the mutational exposures of three signature. The results indicated that our unified approach has higher statistical power for detecting differences in exposures for these signatures while controlling the 5% false positive rate. We suspect that the improvement in statistical power is because our unified method explicitly allows for the uncertainty of inferred mutational exposures, while the two-stage method fails to do so since it incorporates only the point estimates of those exposures. In addition, HiLDA provides posterior distributions for each parameter, thereby allowing construction of 95% credible intervals for parameters, and their differences, for example. As expected, this fully parametric approach is then more powerful than nonparametric approaches, which we see particularly when testing for differences in the rarer signatures.

We also note that the two-stage approach can become problematic with regards to controlling the type I error rate in particular scenarios, e.g., when the variances of exposures differ widely between the two groups. In our simulation study, we aimed to emulate the USC data, meaning that the exposure variances were quite similar between groups. Consequently, the Wilcoxon rank-sum test, the second-stage of the TS approach, was able to maintain a type I error of 5%. However, we note that the Wilcoxon rank-sum test is sensitive to differences found in either location or scale parameters of the two distributions being tested, i.e., it is sensitive to changes in both the mean and the variance. Therefore, when the variances change between two groups, the Wilcoxon rank-sum test may indicate statistically significant differences in distributions even when the means have not changed, (i.e., due to the difference in shape parameters rather than a difference between location parameters). In contrast, HiLDA explicitly focuses on detecting differences in means, and is robust to effects such as changes in variance. Consequently, when applying the TS method, one should be wary of interpreting significant results as evidence of a ”difference in means” when using the TS method (as seems to be common Qin et al., 2018; Meier et al., 2018; Cancer Genome Atlas Research Network, 2017). We note that scenarios in which the variance of the estimated exposures differs will be common if the numbers of mutations per tumor varies between the two groups (e.g., when comparing microsatellite instable vs. microsatellite stable colon tumors), leading to an inflated false-positive rate if results from the TS method are interpreted as being evidence of a difference in means. (See Fig. S5 for a specific example of this.) We intend to explore this issue further in a future paper. We also intend to more fully investigate the factors that drive the ability to detect significant difference between groups across a much wider variety of scenarios.

## Conclusion

In conclusion, we developed a unified method, HiLDA, along with an R package, which enables researchers to simultaneously estimate mutational signatures and infer the mean difference in mutational exposures between two groups. The simulation studies demonstrated that HiLDA has higher statistical power for detecting differences in mutational signatures, because it accounts for uncertainty in the exposure estimates. Application of HiLDA to both the USC colon data and the EAC data suggest that future studies may also benefit from using HiLDA, rather than the existing TS method, to better detect the difference in mutational signatures.

##  Supplemental Information

10.7717/peerj.7557/supp-1 Text S1 The likelihood of HiLDA

10.7717/peerj.7557/supp-2 Table S1 The number of somatic mutations in the 32 mutational catalogs from 16 colon tumors in the USC data, along with their associated trunk and branch mutations

10.7717/peerj.7557/supp-3 Figure S1 The HiLDA diagram in plate notation

10.7717/peerj.7557/supp-4 Figure S2 The result of estimating mutational signatures for the USC data, including 16 trunk mutational catalogs and 16 branch mutational catalogs, using different values of K, the number of mutational signatures

10.7717/peerj.7557/supp-5 Figure S3 Mean mutational exposures and 95% credible interval of differences of three mutational signatures by sex, age groups, smoking status and tumor sites

10.7717/peerj.7557/supp-6 Figure S4 The generative process of a mutation in the simulation study

10.7717/peerj.7557/supp-7 Figure S5 Rejection rates of the two-stage method by different variances in two groups with the same means

## Additional Information and Declarations

### Data Availability

The following information was supplied regarding data availability: The code is available at https://github.com/USCbiostats/HiLDA. The USC colon cancer data is available at https://osf.io/a8dzx/. The EAC data is available at https://www.cbioportal.org/study/summary?id=esca_broad and in Tables S14 (direct link: https://media.nature.com/original/nature-assets/ng/journal/v45/n5/extref/ng.2591-S5.xlsx) and S15 (direct link: https://media.nature.com/original/nature-assets/ng/journal/v45/n5/extref/ng.2591-S6.xlsx) in this article: https://www.nature.com/articles/ng.2591.
