# Supplementary figures and images for: HiLDA: a statistical approach to investigate differences in mutational signatures
Source: PeerJ. 2019 Aug 28;7:e7557. doi: 10.7717/peerj.7557 (PMC6717498; doi:10.7717/peerj.7557)

**Figure S1.**

The HiLDA diagram in plate notation.

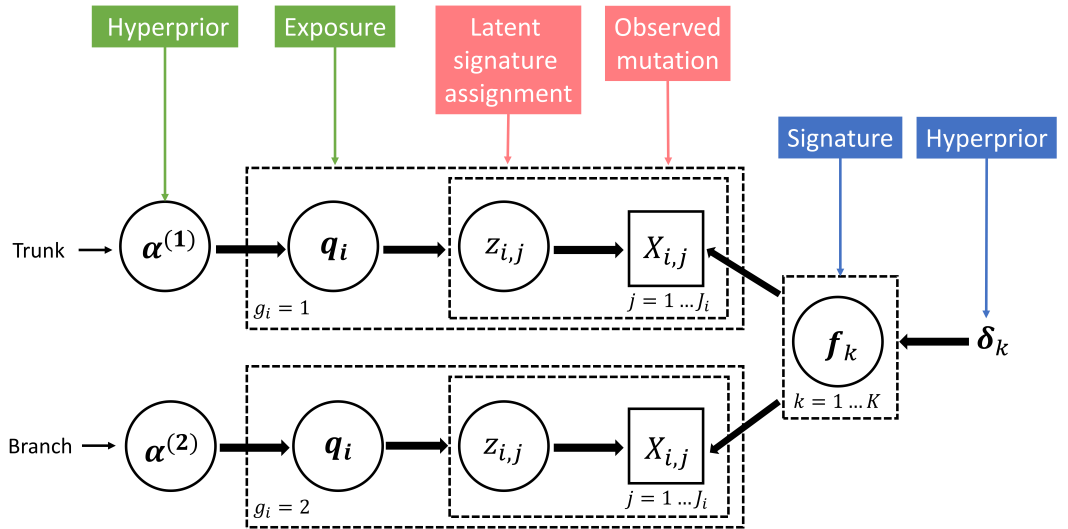

Supplement: Figure S1 [file peerj-07-7557-s003.pdf]
