# Supplementary material for: HiLDA: a statistical approach to investigate differences in mutational signatures
Source: PeerJ. 2019 Aug 28;7:e7557. doi: 10.7717/peerj.7557 (PMC6717498; doi:10.7717/peerj.7557)

Figure S3.

We've included three plots below including: (A) Barplot of mean mutational exposures of three mutational signatures by sex, age groups, smoking status and tumor sites based on the results derived from pmsignature; (B) Same plot as the previous one based on the results derived from HiLDA; (C) 95% credible interval of differences in mutational exposures by sex, age groups, smoking status and tumor sites based on the results derived from the posterior distributions of concentration parameters $\boldsymbol{\alpha}$. Notably, the distributions of mutational exposures over three signatures by smoking status and tumor sites are different from that by sex and age groups, which is resulted from the incomplete information on smoking status and tumor sites for all the patients.

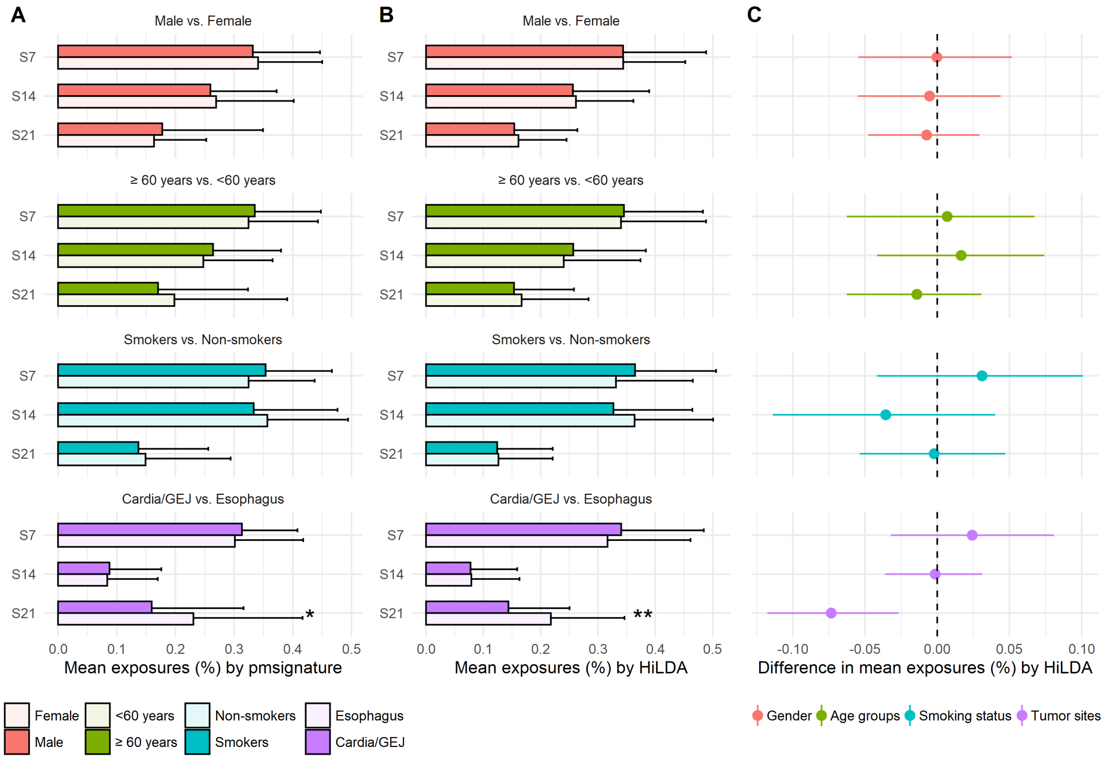

Supplement: Figure S3 [file peerj-07-7557-s005.pdf]
